# Autophagy Regulates the Post-Translational Cleavage of BCL-2 and Promotes Neuronal Survival

**DOI:** 10.1100/tsw.2010.82

**Published:** 2010-05-18

**Authors:** Laura Lossi, Graziana Gambino, Chiara Salio, Adalberto Merighi

**Affiliations:** Dipartimento di Morfofisiologia Veterinaria e Istituto Nazionale di Neuroscienze (INN), Università degli Studi di Torino, Grugliasco (TO), Italy

**Keywords:** BCL-2, post-translational regulation, autophagy, neuronal survival, neurons, cerebellar granule cells, post-natal development, neuronal transfection, organotypic cultures

## Abstract

B-cell lymphoma 2 protein (BCL-2) is one of the more widely investigated anti-apoptotic protein in mammals, and its levels are critical for protecting from programmed cell death. We report here that the cellular content of BCL-2 is regulated at post-translational level along the autophagy/lysosome pathways in organotypic cultures of post-natal mouse cerebellar cortex. Specifically this mechanism appears to be effective in the cerebellar granule cells (CGCs) that are known to undergo massive programmed cell death (apoptosis) during post-natal maturation. By the use of specific agonists/antagonist of calcium channels at the endoplasmic reticulum it was possible to understand the pivotal role of calcium release from intracellular stores in CGC neuroprotection. The more general significance of these findings is supported by a very recent study Niemann-Pick transgenic mice.

Proteins of the BCL-2 family are central regulators of apoptosis. Although they are known to act primarily at the mitochondrial membrane, a pool of the cellular BCL-2 is also linked to the smooth endoplasmic reticulum (SER). The antiapoptotic BCL-2 family of proteins, among which the founding member is BCL-2, appear to function, at least in part, by interacting with and antagonizing their proapoptotic counterparts[1]. Therefore, the balance between these two groups of BCL-2 proteins is crucial to cell survival or death to an extent that any kind of deregulation has been implicated in numerous pathological conditions[2]. Although the precise mechanisms still remain elusive, it is thus conceivable that the BCL-2 cellular levels are subjected to a very strict control. This can be achieved at different steps of protein synthesis, not only during transcription, but also, once translation is completed, by post-translational regulation, i.e., the control of the levels of an active protein inside the cell, either by means of reversible or irreversible events. Reversible events are collectively referred to as post-translational modifications. In brief, they consist of the onset of chemical (such as, for example, the addition of functional groups, other proteins or peptides, and changes of the chemical nature of certain amino acids) or structural changes and/or sequestration in specific cellular compartments. Irreversible events consist of proteolysis or autophagy. Proteolysis of unneeded or damaged proteins is carried out along the ubiquitin-proteasome pathway[3], whereas autophagy is a catabolic process involving the degradation of cellular components through the lysosomal machinery[4]. The ubiquitin-proteasome and autophagy-lysosome pathways are both involved in apoptosis, a type of programmed cell death (PCD) that occurs in multicellular organisms and consists of a series of stereotyped biochemical and morphological events, including cell shrinkage and blebbing, loss of cell membrane asymmetry and attachment, chromatin condensation, and DNA fragmentation, eventually leading to death[5].

Although, in general, the proteasome is not required for apoptosis, primary cultures of quiescent and differentiated cells, such as neurons, are prevented from undergoing apoptosis on exposure to proteasome inhibitors. The mechanism for this effect is not clear, but is hypothesized to be specific to cells in the quiescent state. The involvement of the proteasome in apoptosis is primarily indicated by the increase in protein ubiquitination during the first stages of the process[6]. In addition, proteasomes localized to the nucleus have also been observed to translocate to outer membrane blebs during the apoptotic process[7].

There is no conclusive evidence regarding the involvement of autophagy in apoptosis, and a causative relationship between autophagy and cell death has not yet been established in full[8]. Nevertheless, observations of cells with autophagic features in areas undergoing PCD have led to the definition of an additional type of cell death commonly referred to as autophagic (also known as cytoplasmic cell death or type II cell death), with morphological features somehow different from those of apoptosis. It is not known whether autophagic activity in dying cells actually causes death or whether it simply occurs as a process alongside it. However, in many neurological diseases, in certain neuronal cell death pathways, and after neuronal injury, increased numbers of autophagosomes have been detected[9].

We have investigated the role of autophagy in the post-translational cleavage of BCL-2 and, hence, in the regulation of neuronal survival in organotypic cultures of postnatal mouse cerebellum[10], as a model system to study naturally occurring neuronal death (NOND), a form of PCD that occurs during normal development of the nervous system[11]. The postnatal cerebellar cortex undergoes significant structural remodeling mainly as a consequence of the generation, differentiation, migration, and apoptosis of the cerebellar granule cells (CGCs). It was very recently shown that, similarly to isolated CGCs *in vitro*, differentiation in organotypically cultured CGCs maintained under elevated $[K^+]_e$ is blocked at the stage of immaturity, whereas these neurons reach their full maturation in physiological $[K^+]_e$[12]. The process is reversible and by simply switching from physiological to elevated $[K^+]_e$ or *vice versa*, one can easily interfere with the differentiation state of CGCs. Therefore, we have studied the post-translational regulation of the human BCL-2 (hBCL-2) protein fused to enhanced yellow fluorescent protein (EYFP) in these cultures. We have reasoned that the density of EYFP-hBCL-2–expressing CGCs, directly monitored under the fluorescence microscope, could be used to analyze the consequences of manipulating the degree of CGC maturation on the regulation of the fusion protein, and that any modifications reflected those occurring to endogenous BCL-2[13]. Upon switching from physiological to elevated $[K^+]_e$, we have shown that there are no changes in *bcl-2* mRNA after real-time PCR, whereas the levels of the fusion protein and of murine BCL-2 are increased when cultures are maintained in elevated $[K^+]_e$. After using a series of agonists/antagonists for ion channels at the cell membrane or the endoplasmic reticulum (ER), and drugs affecting protein synthesis/degradation, we related the accumulation of BCL-2 to a reduction in post-translational cleavage by autophagy. Neuronal autophagy is regulated in a quite complex fashion by several signals, among which is $[Ca^{2+}]_i$[14]. Our results indicate that the rise in $[Ca^{2+}]_i$ that follows release from ER stores inhibits the autophagic clearance of BCL-2. In keeping with this assumption, if autophagy is stimulated by rapamycin inhibition of mTOR, the effect of elevated $[K^+]_e$ on the density of EYFP-tagged CGCs is abolished.

To further substantiate these findings, we have devised here an additional series of coimmunoprecipitation experiments. Organotypic cultures were obtained from eight postnatal mice and prepared as described previously[10]. After 4 days *in vitro* (DIV), cultures were subdivided into two experimental groups: in the first, they were maintained in physiological $[K^+]_e$, whereas cultures of the second group were kept in elevated $[K^+]_e$. After three additional DIV, cultures were deep frozen in liquid nitrogen and powdered in general lysis buffer. Protein extracts were immunoprecipitated with a rabbit antihuman BCL-2 primary antibody (1:500) that recognizes the N-terminus of the BCL-2 protein (ab 7973; AbCam, U.K.). After electrophoresis in 9% agarose, immunoprecipitates were blotted onto a nitrocellulose membrane. Membranes were immunostained with a mouse antibeclin 1 (BECN 1) primary antibody (1:500) clone g 11 raised against amino acids 1-300 of BECN 1 of human origin (Santa Cruz Biotechnology, U.S.). Secondary antibodies were a goat antimouse IgG-HRP conjugate (Santa Cruz) and a monoclonal antirabbit IgG-HRP conjugate (A-1949; Sigma, U.S.). Immunoreactions were revealed by Western Lightning Chemiluminescence Reagent Plus (PerkinElmer, U.S.).

These experiments show that BECN 1 coimmunoprecipitates with BCL-2 (Fig. 1A). In addition, the levels of BECN 1 are reduced when cultures are kept in elevated $[K^+]_e$ (Fig. 1B). BECN 1 is a BCL-2–interacting protein that promotes autophagy[15] and a component of the phosphatidylinositol-3-kinase complex required for autophagy[16]. Therefore, these data confirm the interaction of BCL-2 and BECN 1 in cultures, and demonstrate that elevated $[K^+]_e$ reduces the level of BECN 1 expression and, hence, the autophagic clearance of BCL-2 in our experimental system.

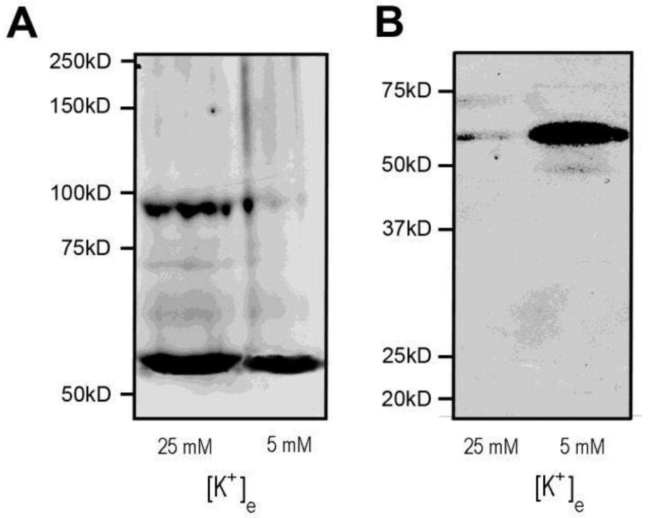

**FIGURE 1.** Localization of BECN 1 in BCL-2 immunoprecipitates (A) or whole protein extracts (B) from postnatal mouse cerebellar organotypic cultures. (A) BECN 1 coimmunoprecipitates with BCL-2 approximately the 85-kD band. The band at approximately 65 kD corresponds to immunoprecipitated IgGs recognized by the secondary antibody. (B) Expression of BECN 1 is drastically reduced under elevated $[K^+]_e$ in comparison to physiological $[K^+]_e$.

Autophagy has been specifically linked to ERK activation in rat CGCs when switching from elevated to physiological $[K^+]_e$. Surprisingly, blocking of ERK did not interfere with the post-translational accumulation of BCL-2 in mouse CGCs[13], although the mechanisms of neuronal death involving ERK may be different between mouse and rat. However, when ERK is blocked, the density of EYFP-hBCL-2–tagged CGCs under elevated $[K^+]_e$ normalized to the mean cell density under physiological $[K^+]_e$ is significantly reduced. This indicates that although not directly affecting the post-translational accumulation of BCL-2, a sustained inhibition of ERK (and hence of autophagy) has a detrimental effect on survival both under mature and immature status. Upstream inducers of ERK have also been hypothesized to play a role in neuronal death[17]. Additional work will be necessary to address this issue fully, but our studies strongly suggest that $[Ca^{2+}]_i$ regulates the post-translational levels of BCL-2 in CGCs through the activation of the CAMK/ERK pathway, consistently with the observations carried out in other types of neurons[18]. In addition, Western blot experiments demonstrate that $[Ca^{2+}]_i$ also regulates the levels of BECN 1. In brief, these results indicate autophagy as an important mechanism for regulation of BCL-2 levels in neurons. As mentioned earlier, apoptosis is the most common aspect that NOND assumes during postnatal maturation of the cerebellar cortex *in vivo*, but autophagy has also been shown to occur in CGCs[19]. In keeping with these observations, previous studies demonstrated that autophagy is constitutively active in neurons and required for neuronal survival[20], although its role in the post-translational regulation of cellular BCL-2 levels was not previously investigated.

The implications of our findings for neuroprotection may be of relevance for a better understanding of the pathogenesis of certain neurodegenerative diseases. For example, earlier studies have shown that severe loss of neurons/terminals in Niemann-Pick Type C1-deficient (Npc1) mice is evident largely in the cerebellar Purkinje neurons[21]. At present, the cell death mechanism remains unclear as events related to both apoptosis and autophagy have been identified in Npc1 mouse brains. Detection of terminal deoxynucleotidyl transferase dUTP nick-end labeling-positive and active caspase-3-immunoreactive Purkinje cells is consistent with cell death being due to apoptosis. In keeping with these results, a very recent study has observed cleaved caspase-3 and Fluoro-Jade C-positive Purkinje neurons in the cerebellum of Npc1 mice[22]. In addition, when the ultrastructural features of cerebellar Purkinje neurons in wild-type and Npc1 mice were compared by using conventional electron microscopy, transgenic mice displayed an evident shrinkage of these neurons that possessed a very intense electron density. The cell body contained severe cytoplasmic vacuolization and a dark nucleus, and, very interestingly, the number and area occupied by secondary lysosomes with heterogeneous dark content and numerous concentric lamellar bodies were increased. Altogether, the ultrastructural alterations of the Purkinje neurons are typical of autophagy and indicate an increase of lysosomal activity. This was confirmed by the increase of the autophagy markers LC3-II and BECN 1 in Npc1 mice compared with controls. Given that antiapoptotic strategies, such as overexpression of BCL-2 or treatment with minocycline (that acts by increasing BCL-2 levels and inhibiting caspase 3), failed to protect neurons in Npc1 mice, we have analyzed the cellular distribution of BCL-2 in Purkinje neurons of these mice, since an obvious possibility deriving from our observations in cultures was that protein levels become drastically reduced as a consequence of an intense autophagic demolition. By the use of the anti–BCL-2 primary antibody described above and postembedding immunogold labeling procedure, we show here that the BCL-2 protein is highly concentrated within secondary lysosomes in Npc1 Purkinje neurons (Fig. 2).

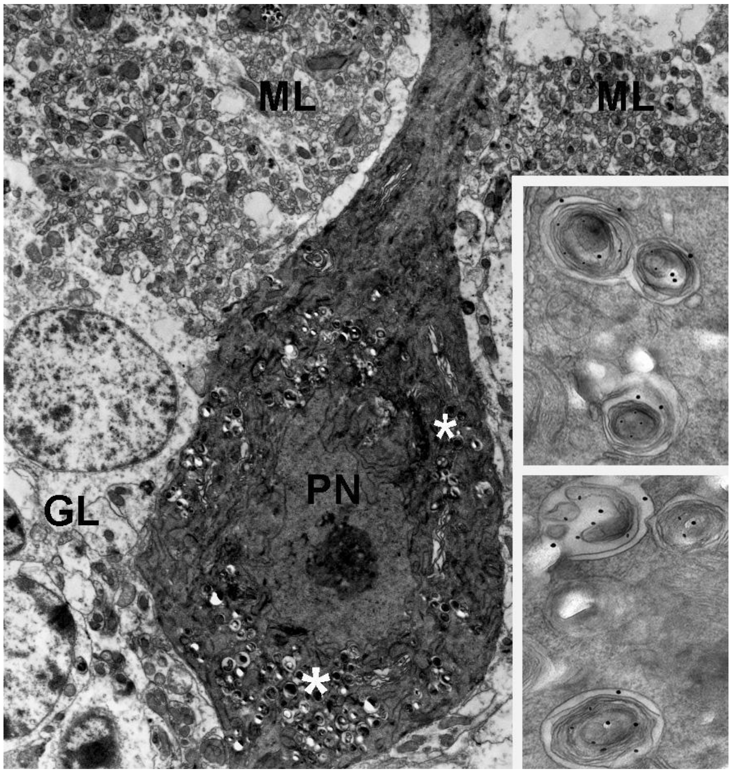

**FIGURE 2.** Purkinje neuron in the cerebellum of Npc1 mouse. Note the accumulation of secondary lysosomes in the cell body. The areas marked by the asterisks are enlarged in the inserts. Note that secondary lysosomes contain gold particles indicative of BCL-2 immunoreactivity. Abbreviations: GL = granular layer of the cerebellar cortex; ML = molecular layer of the cerebellar cortex; PN = Purkinje neuron. Original magnification ×6,000; inserts ×15,000.

This observation unequivocally demonstrates that BCL-2 is sequestered in lysosomes to undergo autophagy in Npc1 mice. Thus, it is conceivable that the lack of neuroprotection by BCL-2 overexpression or minocycline simply reflects a still-maintained imbalance between synthesis and autophagy, although the first was experimentally enhanced by genetic or pharmacological manipulation.

It is also possible that the post-translational regulation of BCL-2 levels by autophagy shows a certain degree of cellular specificity or that redundant apoptotic mechanisms exist in Niemann-Pick pathology.

All data reported above converge to demonstrate an intervention of the autophagic pathway in the regulation of survival of central neurons. A more general understanding of the role of BCL-2 post-translational regulation by autophagy in neurons will no doubt provide further insight on the mechanism of neuroprotection.

## ACKNOWLEDGMENTS

The original work described here was funded by grants of the Italian MiUR to LL (Fondi PRIN 2007).
